# Plasma Aβ as a biomarker for predicting Aβ-PET status in Alzheimer’s disease：a systematic review with meta-analysis

**DOI:** 10.1136/jnnp-2021-327864

**Published:** 2022-03-03

**Authors:** Lizhen Cheng, Wei Li, Yixin Chen, Yijia Lin, Beiyun Wang, Qihao Guo, Ya Miao

**Affiliations:** Department of Geriatrics, Shanghai Jiao Tong University Affiliated Sixth People's Hospital, Shanghai, China

**Keywords:** Alzheimer's disease, amyloid, pet

## Abstract

**Objective:**

Amyloid-β positron emission tomography (Aβ-PET) scan has been proposed to detect amyloid-β (Aβ) deposition in the brain. However, this approach is costly and not ideal for the early diagnosis of Alzheimer’s disease. Blood-based Aβ measurement offers a scalable alternative to the costly or invasive biomarkers. The aim of this study was to statistically validate whether plasma Aβ could predict Aβ-PET status via meta-analysis.

**Methods:**

We systematically searched for eligible studies from PubMed, Embase and Cochrane Library, which reported plasma Aβ levels of amyloid-β positron emission tomography-positive (PET (+)) and amyloid-β positron emission tomography-negative (PET (−)) subjects. We generated pooled estimates using random effects meta-analyses. For any study that has significant heterogeneity, metaregression and subgroup analysis were further conducted. Publication bias was appraised by funnel plots and Egger’s test.

**Results:**

16 studies with 3047 participants were included in the meta-analysis. Among all the enrolled studies, 10 studies reported plasma Aβ40 values, while 9 studies reported plasma Aβ42 values and 13 studies reported Aβ42/Aβ40 ratio. The pooled standardised mean difference (SMD) was 0.76 (95% CI −0.61 to 2.14, p=0.28) in the plasma Aβ40 values group. Plasma Aβ42 values group has a pooled SMD of −0.60 (95% CI −0.80 to −0.41, p<0.0001). In the plasma Aβ42/Aβ40 ratio group, the pooled SMD was −1.44 (95% CI −2.17 to −0.72, p<0.0001).

**Conclusion:**

Plasma Aβ40 values might not distinguish between PET (+) and PET (−) people. However, plasma Aβ42 values and plasma Aβ42/Aβ40 ratio could be served as independent biomarkers for predicting Aβ-PET status.

## Introduction

Alzheimer’s disease (AD), a neurodegenerative disorder, is pathologically characterised by the abnormal accumulation of amyloid-β (Aβ) and hyperphosphorylation of tau. Aβ deposition occuring decades before the onset of clinical symptoms of AD is the first detectable pathological hallmark.^1 2^ Up to now, there is no effective therapy to cure AD as many clinical trials of pharmacological treatment to improve cognitive outcome failed.^3 4^ Providentially, long presymptomatic stage of AD makes it possible to intervene in the early stage with disease-modifying therapy. Therefore, characterising and identifying the early stages of AD through Aβ pathology detection are of great significance.

Currently, Aβ pathology can be identified in vivo with amyloid-β positron emission tomography (Aβ-PET) scans or through altered biomarker levels in the cerebrospinal fluid (CSF).^5–7^ However, Aβ-PET scans are quite costly and not universally accessible in clinical practice, which hampers its feasibility. CSF analysis may be significantly cheaper, but its applicability for periodic population assessment is reduced by implementing lumbar puncture. Given the aforementioned reasons, it is urgently needed to accurately reflect AD pathological processes by blood-based biomarkers which are low-cost, accessible and less invasive.

Recently, growing evidence has accumulated to investigate the potential value of plasma Aβ (Aβ42, Aβ40, Aβ42/Aβ40 ratio, etc) as a screening tool for brain Aβ-PET positivity.^8–10^ Li *et al* 11 proved plasma Aβ42/Aβ40 ratio was associated with Aβ-PET status, with an area under the curve (AUC) of 0.77 (95% CI 0.66 to 0.87). However, an opposite result was reported by Vogelgsang *et al* 12 that the plasma Aβ42/40 ratio did not differ between amyloid-β positron emission tomography-positive (PET (+)) and amyloid-β positron emission tomography-negative (PET (−)) patients. It has cast doubts on the reliability of blood-based Aβ biomarkers predicting Aβ-PET status as different studies have not reached a unanimous conclusion. Since there was no comprehensive meta-analyses of plasma Aβ diagnostic performance until now, it is currently unclear whether plasma Aβ biomarkers can be used as independent prognostic tools to detect AD pathology. In addition, it still remains to be characterised which type of plasma Aβ isoform is suitable for predicting Aβ-PET status accurately.

Under this condition, this study statistically evaluate whether plasma Aβ could predict Aβ-PET status via meta-analysis. The specific purpose of this review was to quantitatively determine (1) what are the differences in plasma Aβ markers between Aβ-PET (+) and Aβ-PET (−) people, (2) whether the plasma Aβ can be used as an independent biomarker for predicting Aβ-PET status and (3) which type of plasma Aβ isoform is suitable for predicting Aβ-PET status accurately.

## Methods

### Data sources

This study followed the Preferred Reporting Items for Systematic Reviews and Meta-Analyses guidelines. Studies on patients assessed with Aβ-PET scans and plasma Aβ were enrolled in this meta-analysis by searching online databases, including Embase, PubMed and Cochrane Library. There were no publish time restrictions on the paper searching. The searching was concluded on 12 July 2021. To ensure that recent studies that may fit the inclusion/exclusion criteria are captured, the search string will be rerun in above three databases in the time window between the completion date of the review and before the completion of the final analysis. Furthermore, the bibliographies of retrieved studies and of any previous reviews will also be examined to identify any additional studies for inclusion. A full list of search terms is included in the online supplemental material 1. The study protocol is available online (https://www.crd.york.ac.uk/prospero/).

10.1136/jnnp-2021-327864.supp1 Supplementary data

### Study selection

The articles were selected based on the following inclusion and exclusion criteria:

Inclusion criteria

1. Studies include the descriptions of specific methods for Aβ-PET and plasma Aβ measurements.
2. Studies include definite groups between PET (+) and PET (−) subjects and their corresponding plasma Aβ values, so that they can be compared.
3. Peer-reviewed manuscripts written in English or translated from their original language of publication to English.
4. Studies of human participants.
5. Studies that provide the mean, SD or SE or CI on plasma Aβ levels.

Exclusion criteria

1. Study subjects had a history of neurological, psychiatric or any systemic disease that could affect cognitive functions (eg, stroke, depression, alcoholism and drug abuse).
2. Review articles, conference papers and case reports are excluded.
3. Studies that do not provide the mean, SD or SE or CI on plasma Aβ levels.

LC, WL, YC and YL were involved in this meta-analysis for screening each record and each report retrieved, independently. If any discrepancies were identified, these were resolved through discussion.

### Data extraction and quality assessment

The data extracted include the primary author for the paper, year the paper was published, sample size, age, Mini-Mental State Examination (MMSE) or Montreal Cognitive Assessment scores, ethnicity, gender, years of education, Aβ-PET tracer agents, methods of measuring plasma Aβ, means and SD, or SE or CI of plasma Aβ levels. If only 95% CI were reported, SE was calculated using the formula SE=(upper bound–mean)/1.96. If only SEs were reported, SD was calculated using the formula SD= √n ×SE.

LC, WL, YC, YL, BW and YM were involved in reviewing abstracts and full texts of each study, independently. Data were double extracted by LC, WL and YC. Further investigation was conducted to determine any duplicate studies/data sets used by considering all of the relevant information provided within the paper, in addition to contacting all authors. Missing data were managed by accessing supplementary material and by contacting all authors. If there were any discrepancies, it will be resolved through discussion, with reference to the manuscript and, if necessary, contacting with the corresponding author of the manuscript.

Quality assessment of included studies was performed using the quality assessment of diagnostic accuracy studies (QUADAS-2) tool.^13^ LC, WL and YC took apart in quality assessment independently and discrepancies were resolved with a fourth reviewer (YL).

### Data syntheses

The data syntheses were conducted using R software V.4.0.2. Effect size was measured using Hedges’ g to correct for small sample size.^14^ A random-effects model was used to calculate the pooled mean effect size, as we wished to make an unconditional inference beyond the included studies.^15^ The heterogeneity analysis was evaluated with I^2^ index to assess the consistency between trials. For any study that has significant heterogeneity, metaregression analysis and subgroup analysis were further conducted. Publication bias was assessed by funnel plots and Egger’s test.^16^ For all tests, a p value of <0.05 was deemed significant.

## Results

### Literature search

**Figure 1:**
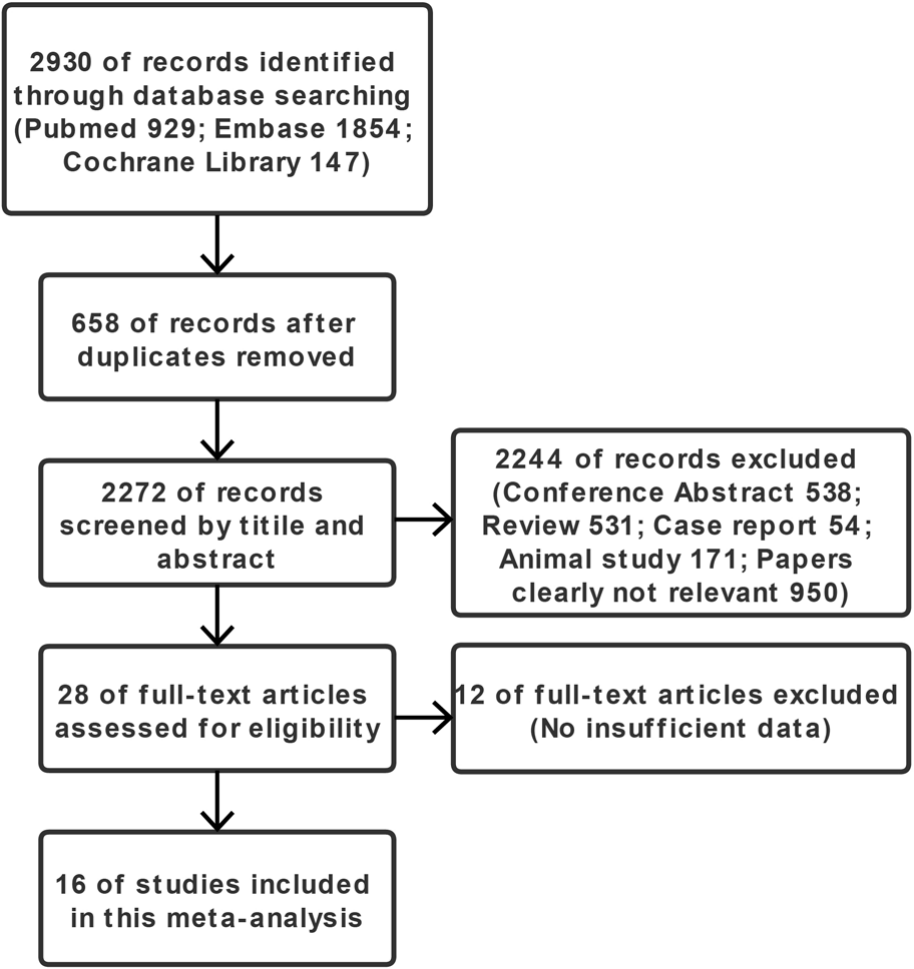
Flowchart of the meta-analysis.

The search process is presented in figure 1. A total of 2930 potentially relevant citations were initially identified. After first round of screening based on titles and abstracts, 28 articles remained for further evaluation. After examining those articles in more detail, 12 articles were excluded as they did not provide sufficient data. In total, 16 articles were included.

### Study characteristics

A total of 16 studies including 3047 subjects were enrolled in this meta-analysis: 1749 PET (−) subjects and 1298 PET (+) subjects. The basic information is shown in tables 1 and 2. Among all included studies, 10 studies reported plasma Aβ40 values; 9 studies reported plasma Aβ42 values; and 13 studies reported Aβ42:Aβ40 ratio. Additionally, Doecke *et al* 17 took plasma samples at three separate time points, which were months 18, 36 and 54. We included the data at month 18 time point in this meta-analysis. Meanwhile, Tosun *et al* 10 divided the participants into two groups, which were the cognitive unimpaired group and the cognitive impairment group. We included both groups with different cognitive conditions in this meta-analysis. In addition, two studies provided the data of plasma Aβ40/Aβ42 ratio and one study reported plasma Aβ oligomerisation (OAβ) in predicting Aβ-PET status. In view of the insufficient number of articles, we did not further analyse them.

**Table 1:** Characteristics of studies used for analysis

Study	Subjects (N)	Dignosis	Main Ethnicity	Male (N)	Female (N)	Age (mean+SD)	Education years (mean+SD)	MMSE (mean+SD)
Chatterjee *et al* 2019^36^	PET (−)=63, PET (+)=32	SMC	White	PET (−)=19, PET (+)=13	PET (−)=44, PET (+)=19	PET (−)=77.65±5.62, PET (+)=79.50±5.32	Not available	PET (−)=28.51±1.15, PET (+)=28.72±1.11
Doecke *et al* 2020^17^	PET (−)=99, PET (+)=77 (18 m)	CN, MCI, AD	White	PET (−)=46, PET (+)=44 (18 m)	PET (−)=53, PET (+)=33 (18 m)	PET (−)=72.7±6.9, PET(+)=75±7.4 (18 m)	Not available	Not available
Kaneko *et al* 2014^37^	PET (−)=22, PET (+)=40 (CN=11)	CN, MCI, AD	Asian	PET (−)=8, PET (+)=19	PET (−)=14, PET (+)=21	PET (−)=72.1±2.9 (CN), PET (+)=75.2±4.7 (all), PET (+)=73.5±4.7 (CN), PET (+)=75.7±4.0 (MCI), PET (+)=76.0±5.0 (AD)	PET (−)=11.6 ± 2.2 (CN); PET (+)=12.0± 2.8 (CN); PET (+)=12.2± 3.2 (MCI); PET (+)=11.4± 2.4 (AD)	PET (−)=28.5±1.5 (CN), PET (+)=28.5±1.3 (CN), PET (+)=26.9±1.4 (MCI), PET (+)=21.6±3.9 (AD)
									Li 2019^11^	PET (−)=36, PET (+)=48	CN, MCI, AD	Asian	Not available	Not available	CN=61.78±10.52, MCI=64.60±9.37, AD=68.39±9.65	CN=12.50±4.95, MCI=10.76±4.47, AD=10.20±4.20	Not available
Lin 2019^38^	PET (−)=30, PET (+)=22	aMCI, mild AD	Asian	PET (−)=19, PET (+)=11	PET (−)=11, PET (+)=11	PET (−)=71.9 ± 9.7, PET (+)=72.1±7.6	PET (−)=11.0±3.6, PET (+)=12.0±4.3	PET (−)=27.0±2.2, PET (+)=24.0±2.7
Nakamura *et al* 2018^39^	PET (−)=186, PET (+)=187	CN MCI, AD	White, Asian	PET (−)=89, PET (+)=93	PET (−)=97, PET (+)=94	PET (−)=73.64±5.68, PET (+)=74.57±5.39	Not available	Not available
Palmqvist *et al*2019^40^	PET (−)=226, PET (+)=151	CN, MCI	White	PET (−)=104, PET (+)=85	PET (−)=122, PET (+)=66	PET (−)=71.8±5.6, PET (+)=72.6±5.0	Not available	PET (−)=28.5±1.5, PET (+)=27.8±1.6
Park 2017^41^	PET(−)=253, PET (+)=100	CN, MCI, AD	Asian	PET (−)=95, PET (+)=38	PET (−)=158, PET (+)=62	PET (−)=69.94±0.5, PET (+)=73.00±0.7	PET (−)=10.65±0.3, PET (+)=10.82±0.5	Not available
Pérez-Grijalba *et al* 2019^42^	PET (−)=41, PET (+)=18	CN, MCI	Spanish	PET (−)=23, PET (+)=9	PET (−)=18, PET (+)=9	PET (−)=71.6±4.11, PET (+)=75.2±5.65	PET (−)=12.33±3.96, PET (+)=10.56±4.44	Not available
Schindler *et al* 2019^43^	PET (−)=115, PET (+)=43	CN	White	PET (−)=43, PET (+)=13	PET (−)=72, PET (+)=30	PET (−)=60.8±6.7, PET (+)=71.4±6.8	PET (−)=15.9±2.2, PET (+)=15.2±3.2	PET (−)=29.4±0.8, PET (+)=29.0±1.6
Vergallo 2019^34^	PET (−)=203, PET (+)=74	SMC	White	PET (−)=80, PET (+)=27	PET (−)=123, PET (+)=47	PET (−)=76.6±3.4, PET (+)=77.3±3.2	Not available	Not available
Wang *et al* 2020^8^	PET (−)=28, PET (+)=18	CN, aMCI, AD	Asian	PET (−)=17, PET (+)=7	PET (−)=11, PET (+)=11	PET (−)=70.1±10.3, PET (+)=71.8±7.92	PET (−)=11.8±3.6, PET (+)=13.2± 4.1	PET (−)=27.11±2.74, PET (+)=24.11±2.70
Verberk *et al* 2020^44^	PET (−)=76, PET (+)=176	SCD, MCI, AD	White	PET (−)=49, PET (+)=89	PET (−)=27, PET (+)=87	PET (−)=61±9, PET (+)=63±7	Not available	PET (−)=27±2, PET (+)=23 ± 4
West *et al* 2021^45^	PET (−)=253, PET (+)=161	CN, MCI to AD, AD	White	PET (−)=92, PET (+)=79	PET (−)=161, PET (+)=82	PET (−)=67.7±8.1, PET (+)=73.6±7.4	PET (−)=16.3±2.4, PET (+)=16.1±2.5	PET (−)=29.4±1.6, PET (+)=26.2±4.5
Tosun *et al* 2021^10^	PET (−)=50, PET(+)=37 (CU); PET (−)=40, PET (+)=46 (CI)	CU, CI	White	PET (−)=25, PET (+)=35 (CU); PET (−)=19, PET (+)=22 (CI)	PET (−)=25, PET (+)=12 (CU); PET (−)=21, PET (+)=24 (CI)	PET (−)=71.9±6.1, PET (+)=75.3±5.2 (CU); PET (−)=70.0±7.9, PET (+)=73.1±6.9 (CI)	PET (−)=16.8±2.6, PET (+)=16.1±2.4 (CU); PET (−)=16.4+2.5, PET (+)=16.0+3.0 (CI)	PET (−)=29.2±1.0, PET (+)=28.9±1.0 (CU); PET (−)=28.5±1.3, PET (+)=27.6±2.0 (CI)
									Pyun *et al* 2021^35^	PET (−)=28, PET (+)=68	SCD, MCI, AD, OND	Asian	PET (−)=13, PET (+)=29	PET (−)=15, PET (+)=39	Not available	Not available	Not available

aMCI, amnesic mild cognitive impairment; CI, cognitive impaired; CN, normal control; CU, cognitive unimpaired; MCI, mild cognitive impairment; MMSE, Mini-Mental State Examination; OND, other neurodegenerative disease; PET (−), amyloid-β positron emission tomography-negative; PET (+), amyloid-β positron emission tomography-positive; SCD, subjective cognitive decline; SMC, subjective memory complainer.

**Table 2:** Key details: data acquisition of studies used for analysis

Study	Plasma Aβ40 levels	Plasma Aβ42 levels	Plasma Aβ42/Aβ40 ratio	Other Aβ measures	Method	PET tracer
Chatterjee *et al* 2019^36^	PET (−)=307.44±54.16, PET (+)=332.82±73.71	PET (−)=16.01±3.74, PET (+)=15.71±3.48	PET (−)=0.052±0.008, PET (+)=0.047±0.005	Not available	ELISA	FBB
Doecke *et al* 2020^17^	Not available	Not available	PET (−)=0.092±0.027, PET (+)=0.075±0.02 (18 m)	Not available	ELISA	PiB
Kaneko *et al* 2014^37^	Not available	PET (−)=0.21±0.072, PET (+)=0.14±0.065, PET (+) (CN)=0.14±0.051	PET (−)=0.011±0.005, PET (+)=0.007±0.003, PET (+) (CN)=0.007±0.003	Not available	IP-MS	PiB
Li 2019	PET (−)=219.14±116.15, PET (+)=221.76±109.53	PET (−)=10.91±5.44, PET (+)=9.01±4.87	PET (−)=0.0566±0.0231, PET (+)=0.0425±0.0203	Not available	Simoa	PiB
Lin 2019^38^	PET (−)=49.1±7.3, PET (+)=50.9±7.7	PET (−)=17.6±3.3, PET (+)=16.3±2.3	PET (−)=0.374±0.117, PET (+)=0.334±0.109	Not available	IMR	AV45
Nakamura *et al* 2018^39^	PET (−)=8.733±2.044, PET(+)=8.280±1.820	PET (−)=0.379±0.095, PET (+)=0.291±0.067	PET (−)=0.044±0.006, PET (+)=0.035±0.004	PET(-)=23.373±3.313, PET(+)=28.729±3.060 (Aβ40/Aβ42 ratio)	IP–MS	PiB, FLUTE, AV45
Palmqvist *et al* 2019^40^	PET (−)=485±71, PET (+)=483±73	PET (−)=32.7±4.7, PET (+)=29.9±4.7	PET (−)=0.0680±0.0077, PET (+)=0.0622±0.0078	Not available	ELISA	FLUTE
Park 2017^41^	PET (−)=118.70±2.09, PET (+)=136.60±3.37	Not available	PET (−)=0.36±0.01, PET (+)=0.30± 0.01	Not available	xMAP	PiB
Pérez-Grijalba *et al* 42 2019	Not available	Not available	PET (−)=0.1329 ±0.0208, PET (+)=0.0997±0.0197	Not available	ELISA	PiB
Schindler *et al* 2019^43^	Not available	Not available	PET (−)=0.128±0.009, PET (+)=0.115±0.006	Not available	IP–MS	PiB, AV45
Vergallo 2019^34^	PET (−)=301.9± 87.8, PET (+)=295.5±75.4	PET (−)=18.4±5.8; PET (+)=15.1±4.0	Not available	PET (−)=16.7±5.2, PET (+)=19.4±3.3 (Aβ40:Aβ42 ratio)	ELISA	AV45
Wang *et al* 2020^8^	PET (−)=48.57±7.71, PET (+)=51.84±6.75	Not available	Not available	Not available	IMR	AV45
Verberk *et al* 2020^44^	PET (−)=165±30, PET (+)=157±28	PET (−)=27±6, PET(+)=23±6	PET (−)=0.17±0.03, PET (+)=0.14±0.03	Not available	Simoa	FBB, PiB, FLUTE
West *et al* 2021^45^	PET (−)=440.435±81.870, PET (+)=452.325±103.933	PET (−)=44.477±8.637, PET (+)=40.421±9.698	PET (−)=0.101±0.010, PET (+)=0.090±0.010	Not available	LC-MS/MS	PiB, FBB, AV45
Tosun *et al* 2021^10^	Not available	Not available	PET (−)=0.12±0.01, PET (+)=0.11±0.01 (CU); PET (−)=0.13±0.01, PET (+)=0.11±0.009 (CI)	Not available	IP–MS	AV45
							Pyun *et al* 2021^35^	Not available	Not available	Not available	PET (−)=0.67±0.21, PET (+)=0.89±0.17 (OAβ)	ELISA	FBB, AV45, FLUTE

AV45, 18F-florbetapir; Aβ, amyloid-β; ELISA, enzyme linked immunosorbent assay; FBB, 18F-florbetaben; FLUTE, 18F-flutemetamol; IMR, immunomagnetic reduction; IP-MS, immunoprecipitation-mass spectrometry; LC-MS/MS, high-throughput, liquid chromatography–tandem mass spectrometry; OAβ, Aβ oligomerisation; PET (+), amyloid-β positron emission tomography-positive; PET (−), amyloid-β positron emission tomography-negative; PiB, [11C] Pittsburgh compound B; Simoa, single-molecule array; xMAP, flexible multi-analyte profiling.

The results of quality assessment are summarised in online supplemental material 2.

10.1136/jnnp-2021-327864.supp2 Supplementary data

### Results of pooled effect size

**Figure 2:**
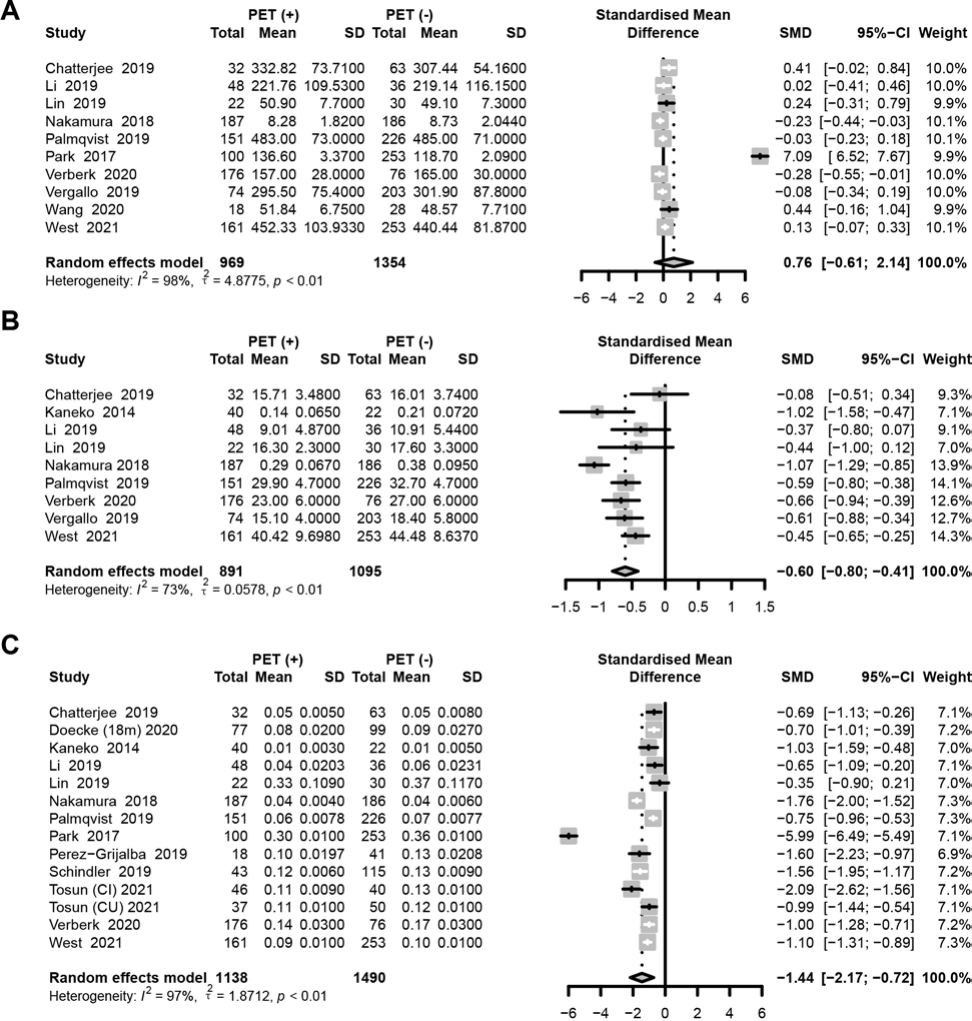
Outcome of meta-analysis of the Aβ40 group (A), p=0.28; Aβ42 group (B), p<0.0001; and the Aβ42/Aβ40 ratio group (C), p<0.0001. SMD, standardised mean difference.

We first meta-analysed data on plasma Aβ40 values in PET (−) subjects and PET (+) subjects (figure 2A). The pooled standardised mean difference (SMD) was 0.76 (95% CI −0.61 to 2.14, random-effects model). The overall effect was not significant (p=0.28), which implied there was no statistical distinction in plasma Aβ40 values between PET (+) and PET (−) subjects. In addition, high heterogeneity was found with an I^2^=98%.

Second, we meta-analysed reported data on plasma Aβ42 values (figure 2B). The pooled SMD was −0.60 (95% CI −0.80 to −0.41, random-effects model). The overall effect was tremendously significant (p<0.0001), which meant plasma Aβ42 values could be used as an independent biomarker for predicting Aβ-PET status. Meanwhile, heterogeneity was found with an I^2^=73%.

For Aβ42:Aβ40 ratio, the pooled SMD was −1.44 (95% CI −2.17 to −0.72, random-effects model) (figure 2C). The overall effect was also quite significant (p<0.0001), which indicated that plasma Aβ42:Aβ40 ratio had the ability to be a biomarker for distinguishing people with PET (+) and PET (−) as well. Heterogeneity was large, with an I^2^=97%.

### Results of metaregression analysis

We used mean age, mean MMSE scores and mean education years as moderators in the metaregression models. However, we did not find any of these factors could account for the variance between the various studies (online supplemental materials 3–5).

10.1136/jnnp-2021-327864.supp3 Supplementary data

10.1136/jnnp-2021-327864.supp4 Supplementary data

10.1136/jnnp-2021-327864.supp5 Supplementary data

### Results of subgroup analysis

Considering the heterogeneity of the results, we performed subgroup analysis for the following factors:

1. The characteristics of the study participants included. We conducted subgroup analysis based on the ethnicity background of the study participants in plasma Aβ40, Aβ42 and Aβ42:Aβ40 ratio group, respectively (online supplemental material 6). In the Aβ40 groups, even though the heterogeneity decreased in the white ethnicity subgroup, heterogeneity was still very high in the Asian ethnicity subgroup. In addition, the pooled effect in all subgroups had no statistical significance but was significantly different for different ethnicity backgrounds. In the Aβ42 groups, the heterogeneity also decreased in white ethnicity subgroup but increased in the Asian ethnicity subgroup. Besides, the pooled effect only slightly changed in the different subgroups. In the Aβ42:Aβ40 ratio group, the heterogeneity in each subgroup was all in a high level, and no obvious changes of pooled effect were observed in each subgroup. Additionally, test for subgroup difference was all of no statistical significance in three groups (Aβ40 group: p=0.30, Aβ42 group: p=0.25, Aβ42/Aβ40 ratio group: p=0.27). These results indicated that ethnicity background of the study participants was not a source of heterogeneity.
2. Aβ-PET tracer selection. Different PET tracers were used in the studies, on which we performed further subgroup analysis (online supplemental material 7). In the plasma Aβ40 group, the heterogeneity decreased in 18F-flutemetamol (FLUTE) and AV45 subgroups but increased in the [11C] Pittsburgh compound B (PiB) subgroup. The pooled effect of all subgroup results had no statistical significance but was significantly different for different PET tracers. In the plasma Aβ42 group, the heterogeneity also decreased in FLUTE and AV45 subgroups and increased in the PiB subgroup. Besides, the pooled effect in all subgroups was significant except for the FBB subgroup. In the plasma Aβ42:Aβ40 ratio group, the heterogeneity only decreased in the FLUTE subgroup, and the pooled effect of all subgroups was significant and slightly changed. In addition, test for subgroup difference was all of no statistical significance in the three groups (Aβ40 group: p=0.21, Aβ42 group: p=0.09 and Aβ42:Aβ40 ratio group: p=0.38). These results indicated that Aβ-PET tracer was also not a source of heterogeneity.
3. Selection of plasma Aβ measurement methods. As Aβ isoform in the blood has a low level, different detection methods might have a great impact on the results, and we consequently carried out a subgroup analysis according to the measurement methods (online supplemental material 8). In the plasma Aβ40 group, the heterogeneity decreased in all subgroups. According to pooled effect size, plasma Aβ40 values did not differ between people who were PET (+) and PET (−). However, subgroup analysis showed that when immunoprecipitation-mass spectrometry or flexible multi-analyte profiling (xMAP) was chosen as the plasma Aβ measurement method, the effect was significant. In the plasma Aβ42 group, the heterogeneity also decreased in all subgroups. Besides, when immunomagnetic reduction was used as plasma Aβ measurement method, the effect had no statistical significance. In the plasma Aβ42:Aβ40 ratio group, the heterogeneity decreased in all subgroups as well. Besides, the SMD was significantly different for different plasma Aβ measurement methods. In addition, the results of tests for subgroup difference were statistically significant in the three groups (Aβ40 group: p<0.01, Aβ42 group: p<0.01, Aβ42:Aβ40 ratio group: p<0.01). These results together indicated that the plasma Aβ measurement method was probably the main source of heterogeneity in the three groups.

10.1136/jnnp-2021-327864.supp6 Supplementary data

10.1136/jnnp-2021-327864.supp7 Supplementary data

10.1136/jnnp-2021-327864.supp8 Supplementary data

### Analysis of publication bias

**Figure 3:**
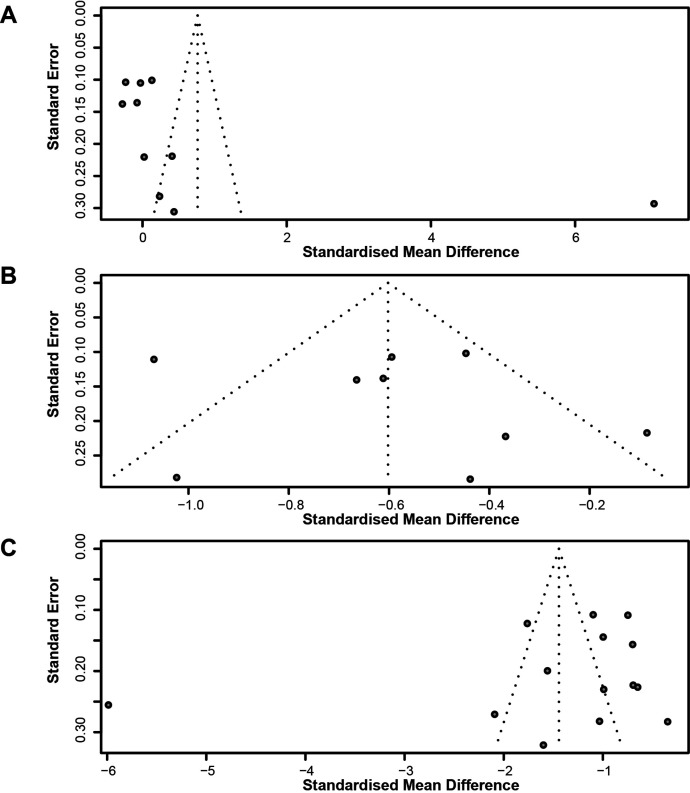
Funnel plots the Aβ40 group (A), the Aβ42 group (B) and the Aβ42:Aβ40 ratio group.

**Figure 4:**
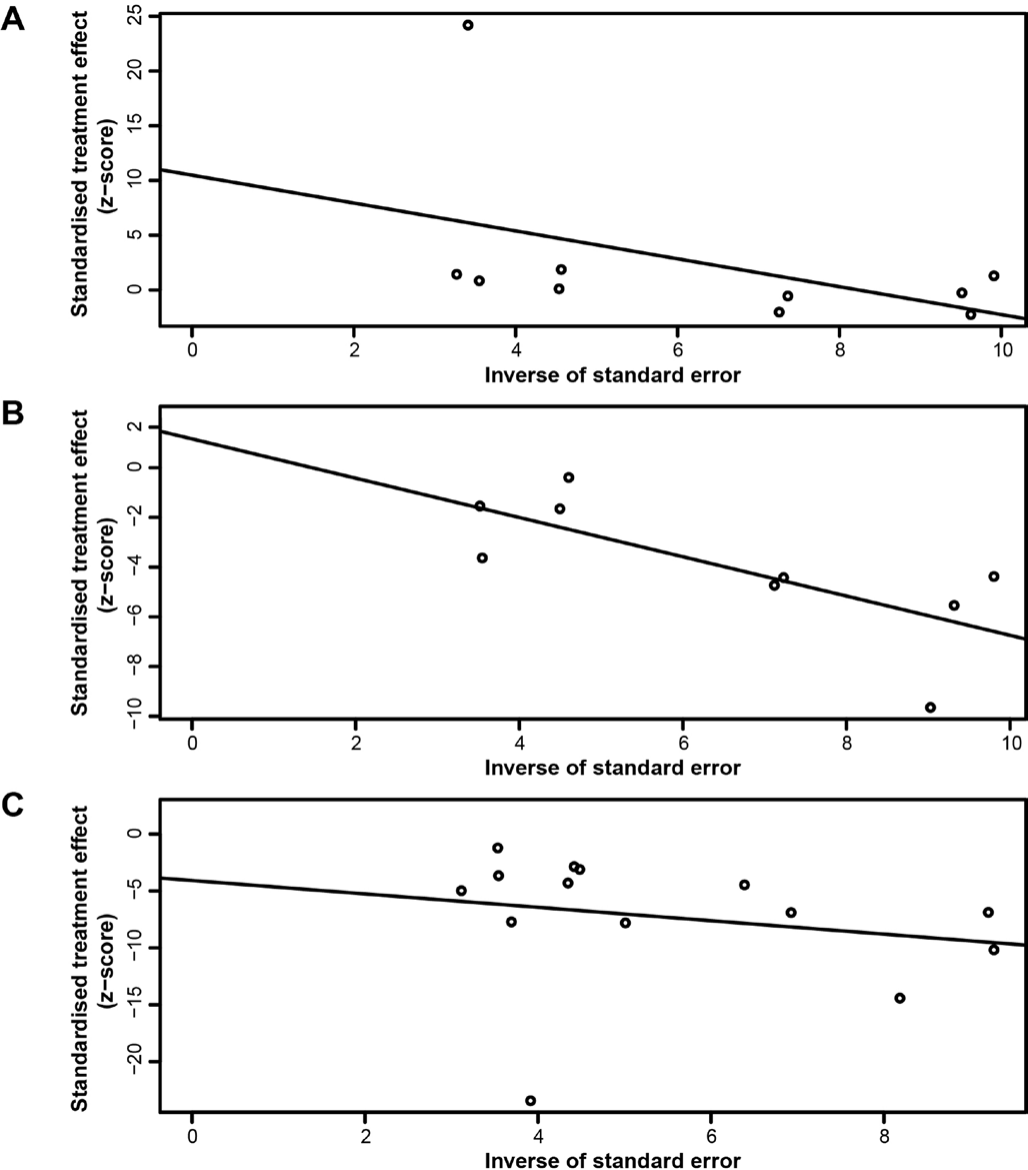
Outcome of Egger’s test for the Aβ40 group (A), t=1.72 and p=0.1234; the Aβ42 group (B), t=0.60 and p=0.5675; and the Aβ42:Aβ40 ratio group (C), t=−0.94 and p=0.3662.

The relative funnel plots are shown in figure 3. Furthermore, Egger’s test indicated the absence of publication bias (figure 4; Aβ40 group: t=1.72 and p=0.1234, Aβ42 group: t=0.60 and p=0.5675, Aβ42:Aβ40 ratio group: t=−0.94 and p=0.3662).

## Discussion

Many studies are working on identifying available and effective AD biomarkers. Aβ accumulation occurs decades before the onset of AD symptoms and is suitable for identifying the early stages of AD. However, CSF analysis and Aβ-PET imaging are not appropriate for screening people at risk of AD development.^18^ Blood-based biomarkers, which are less invasive, cost-effective and procedurally simple, are expected to facilitate critical clinical solutions. In this study, we sought to evaluate the evidence on plasma Aβ predicting Aβ-PET status via meta-analysis. To the best of our knowledge, this is the first meta-analysis providing comprehensive insights into the possibility that plasma Aβ as a biomarker for screening cerebral Aβ deposition of PET.

Aβ is an aggregation-prone and toxic polypeptide with 39–43 residues, derived from the AD proteolysis process of amyloid precursor protein.^19 20^ Among all Aβ isoforms, Aβ40 and Aβ42 are believed to be the most important ones.^21^ Aβ40 and Aβ42 are quite similar in their sequences; the only difference between them is an extra isoleucine and an alanine at the C-terminus of Aβ42.^22^ In this meta-analysis, we figured out plasma Aβ40 values might not differ between PET (+) and PET (−) subjects. However, plasma Aβ42 values were significantly different between the two populations, suggesting that plasma Aβ42 values could be regarded as an independent biomarker for predicting Aβ-PET status. Obviously, compared with plasma Aβ40 values, plasma Aβ42 values seems to be better for the stability and accuracy of reflecting brain Aβ-PET status. In addition, PET (+) subjects showed a marked reduction in plasma Aβ42 values, which is consistent with the result of CSF Aβ42 in AD.^23^ Aβ42 aggregates were the major components of amyloid plaque in the brains of patients with AD.^24^ Aβ42 aggregation formed senile plaques in the brain parenchyma, resulting in lower amounts being secreted to the extracellular space and the CSF. As the blood–brain barrier and the blood–CSF barrier regulate the passage of solutes Aβ42 between blood and the central nervous system (CNS), decreased CSF Aβ42 levels might lead to lower blood Aβ42 levels. However, why is Aβ42 more efficient than Aβ40 in predicting Aβ-PET status? As reported, Aβ42 is highly prone to aggregate amyloid plaque in the brain early, and its oligomers are highly toxic to neurons,^25^ whereas Aβ40 may have antioxidant and antiamyloidogenic effects and predominantly exists in cerebral amyloid angiopathy.^24 26^ In the progression of AD, as a meta-analysis reported, CSF Aβ42 levels are markedly reduced, while Aβ40 levels remain within normal ranges.^27^ This change might be related to the key pathophysiology that Aβ deposited in brain tissue and amyloid plaques in AD largely consists of Aβ42 peptides ending at position.^28^ Postmortem studies have shown that decreased CSF Aβ42 levels are associated with higher plaque counts, and a large number of studies have shown that CSF Aβ42 levels are highly consistent with amyloid PET status, which further support this explanation.^29 30^ In addition, plasma Aβ mainly comes from CNS. The aforementioned reasons explain why plasma Aβ42 values are more related to the Aβ pathology status measured by Aβ-PET compared with plasma Aβ40 values. Then we meta-analysed data on the Aβ42:Aβ40 ratio group; the results indicated that the plasma Aβ42:Aβ40 ratio also could be a biomarker for distinguishing PET (+) and PET (−) subjects. There might be several possible reasons for this. During AD progression, Aβ42 levels are markedly reduced, while Aβ40 levels might stay in a plateau stage. The Aβ42:Aβ40 ratio could play a role in correcting individual differences. A previous meta-analysis^31^ obtained similar results that lower levels of the plasma Aβ42:Aβ40 ratio reflect a process of selective deposition of Aβ42 in the brain as insoluble amyloid plaques, thus predictive of dementia development, while plasma levels of Aβ40 and Aβ42 alone were not significantly associated with either outcome.

Considering the high heterogeneity of the aforementioned results, metaregression analysis and subgroup analysis were further conducted. However, according to metaregression analysis, mean age, mean MMSE scores and mean education years all could not account for the variance between the various studies. We further performed subgroup analysis based on the ethnicity background of the study participants, Aβ-PET tracer and plasma Aβ measurement method selections. According to the results of the subgroup analysis, we found that plasma Aβ measurement methods might be mainly a source of heterogeneity. As we all know, plasma Aβ isoform is in a low level and the hydrophobic nature of Aβ makes the peptide bind to plasma proteins, which could result in epitope masking and other analytical interferences.^32^ Therefore, the method of plasma Aβ measurement is pivotal in practice. De Meyer *et al* 9 quantified plasma Aβ42:Aβ40 ratios with both routinely available ELISA and novel single-molecule array (Simoa) assays and provided a head-to-head comparison of their performances to detect cerebral amyloidosis in a non-demented elderly cohort. They reported that ELISA and Simoa plasma Aβ42/Aβ40 detected cerebral amyloidosis with identical accuracy (ELISA: AUC 0.78, 95% CI 0.72 to 0.84; Simoa: AUC 0.79, 95% CI 0.73 to 0.85), and plasma Aβ levels showed poor agreement between ELISA and Simoa with concentrations of both Aβ42 and Aβ40 measured by Simoa consistently underestimating those measured by ELISA. In a recent study, Janelidze *et al* 33 compared the performance of plasma Aβ42/40 measured using eight different Aβ assays when detecting abnormal brain Aβ status in patients with early AD. The results from two independent cohorts indicated that certain mass spectrometry (MS)-based methods performed better than most of the immunoassays for plasma Aβ42/40 when detecting brain Aβ pathology. A suitable method allows a more accurate measurement of plasma Aβ fluctuation and consequently higher efficiency in screening Aβ-PET status.

Several limitations of this meta-analysis should be considered. In view of the insufficient number of articles, we did not meta-analyse the data of the plasma Aβ40:Aβ42 ratio and plasma oligomeric amyloid-β (OAβ). Vergallo *et al* 34 investigated whether plasma concentrations of the Aβ40:Aβ42 ratio, assessed using Simoa immunoassay, could predict brain Aβ-PET status in a large-scale longitudinal monocentric cohort of older individuals with subjective memory complaints. The receiver operating characteristic curve and machine learning showed a balanced accuracy of 76.5% and 81%, respectively, for the plasma Aβ40:Aβ42 ratio. Additionally, Pyun *et al* 35 reported plasma OAβ could also predict Aβ-PET positivity with high performance, and, when it is combined with age, MMSE score and APOE ε4 status, predictability was improved substantially. It suggests the potential of OAβ as an informative initial stage test in the clinical and research fields of AD. However, confirmation of the role of these two indicators in predicting Aβ-PET status still remains to be investigated. Additionally, according to this meta-analysis, we can only preliminarily confirm that plasma Aβ42 and plasma Aβ42:Aβ40 ratio can distinguish between PET (+) and PET (−) populations, but the specific diagnostic accuracy needs to be further evaluated.

## Conclusion

In conclusion, this meta-analysis provides evidence that plasma Aβ40 values might not distinguish between PET (+) and PET (−) people. However, plasma Aβ42 values and Aβ42/Aβ40 ratio were associated with Aβ-PET status. In the development of relevant research, special attention should be paid to the selection of plasma Aβ measurement methods.

## Data Availability

All data relevant to the study are included in the article or uploaded as supplementary information. All data relevant to the study are included in the article or uploaded as supplementary information. Data are available to qualified investigators on request to the corresponding author.
