# Supplementary material for: Plasma Aβ as a biomarker for predicting Aβ-PET status in Alzheimer’s disease：a systematic review with meta-analysis
Source: J Neurol Neurosurg Psychiatry. 2022 Mar 3;93(5):513–20. doi: 10.1136/jnnp-2021-327864 (PMC9016262; doi:10.1136/jnnp-2021-327864)
Supplement: Supplementary data [file jnnp-2021-327864supp001.pdf]

Search strategies

Search String in Pubmed:

1# Alzheimer's disease OR Alzheimer OR dementia OR AD OR cogniti*

2# A beta OR Aβ OR amyloid

3# PET OR positron emission tomography OR PiB OR Pittsburgh compound B OR AV-45 OR AV45 OR FBP OR florbetapir OR FBB OR florbetaben OR Vizamyl OR FLUTE OR Flutemetamol

4# plasma OR blood OR sanguis

1# AND 2# AND 3# AND 4#

Search String in Embase:

1# Alzheimers disease OR Alzheimer OR dementia OR AD OR cogniti*

2# A beta OR Aβ OR amyloid

3# PET OR positron emission tomography OR PiB OR Pittsburgh compound B OR AV-45 OR AV45 OR FBP OR florbetapir OR FBB OR florbetaben OR Vizamyl OR FLUTE OR Flutemetamol

4# plasma OR blood OR sanguis

1# AND 2# AND 3# AND 4#

Search String in Cochrane Library:

1# Alzheimer's disease OR Alzheimer OR dementia OR AD OR cogniti*

2# A beta OR Aβ OR amyloid

3# PET OR positron emission tomography OR PiB OR Pittsburgh compound B OR AV-45 OR AV45 OR FBP OR florbetapir OR FBB OR florbetaben OR Vizamyl OR FLUTE OR Flutemetamol

4# plasma OR blood OR sanguis

1# AND 2# AND 3# AND 4#
