# Supplementary material for: Plasma Aβ as a biomarker for predicting Aβ-PET status in Alzheimer’s disease：a systematic review with meta-analysis
Source: J Neurol Neurosurg Psychiatry. 2022 Mar 3;93(5):513–20. doi: 10.1136/jnnp-2021-327864 (PMC9016262; doi:10.1136/jnnp-2021-327864)
Supplement: Supplementary data [file jnnp-2021-327864supp002.pdf]

**Supplemental Files 2. The quality of citations.**

| Study | Risk of bias | | | | Applicability concerns | | |
|---|---|---|---|---|---|---|---|
| | **Patient selection** | **Index test** | **Reference standard** | **Flow and timing** | **Patient selection** | **Index test** | **Reference standard** |
| Chatterjee 2019 | ☹ | ☺ | ☺ | ☹ | ☺ | ☺ | ☺ |
| Doecke 2020 | ☹ | ☹ | ☺ | ☹ | ☹ | ☺ | ☺ |
| Kaneko 2014 | ☹ | ☹ | ☺ | ☺ | ☹ | ☺ | ☺ |
| Li 2019 | ☺ | ☹ | ☺ | ? | ☹ | ☺ | ☺ |
| Lin 2019 | ☹ | ☹ | ☺ | ? | ☺ | ☺ | ☺ |
| Nakamura 2018 | ☺ | ☺ | ☺ | ☹ | ☺ | ☺ | ☺ |
| Palmqvist 2019 | ☺ | ☹ | ☺ | ? | ☺ | ☺ | ☺ |
| Park 2017 | ☺ | ☺ | ☺ | ☹ | ☺ | ☺ | ☺ |
| Pérez-Grijalba 2019 | ☹ | ☹ | ☺ | ☺ | ☹ | ☺ | ☺ |
| Schindler 2019 | ☺ | ☺ | ☺ | ? | ☹ | ☺ | ☺ |
| Vergallo 2019 | ☹ | ☺ | ☺ | ☹ | ☺ | ☺ | ☺ |
| Wang 2020 | ☹ | ☹ | ☺ | ☺ | ☺ | ☺ | ☺ |
| Verberk 2020 | ☹ | ☹ | ☺ | ☹ | ☹ | ☺ | ☺ |
| West 2021 | ☹ | ☹ | ☺ | ☹ | ☹ | ☺ | ☺ |
| Tosun 2021 | ☹ | ☹ | ☺ | ☹ | ☹ | ☺ | ☺ |
| Pyun 2021 | ☹ | ☹ | ☺ | ? | ☹ | ☺ | ☺ |

☺Low Risk ☹High Risk ? Unclear Risk
