# Supplementary figures and images for: Plasma Aβ as a biomarker for predicting Aβ-PET status in Alzheimer’s disease：a systematic review with meta-analysis
Source: J Neurol Neurosurg Psychiatry. 2022 Mar 3;93(5):513–20. doi: 10.1136/jnnp-2021-327864 (PMC9016262; doi:10.1136/jnnp-2021-327864)

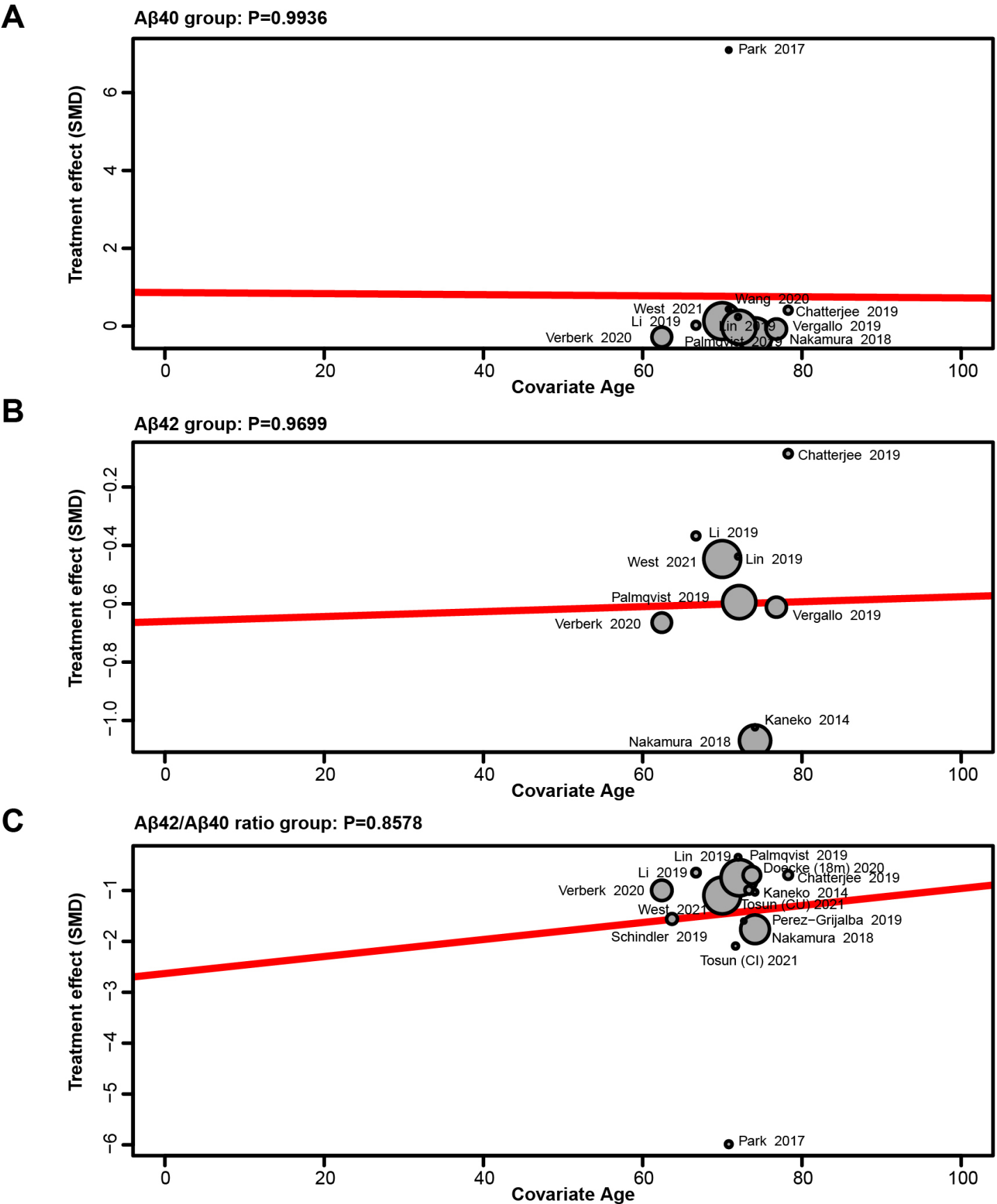

Supplement: Supplementary data [file jnnp-2021-327864supp003.pdf]

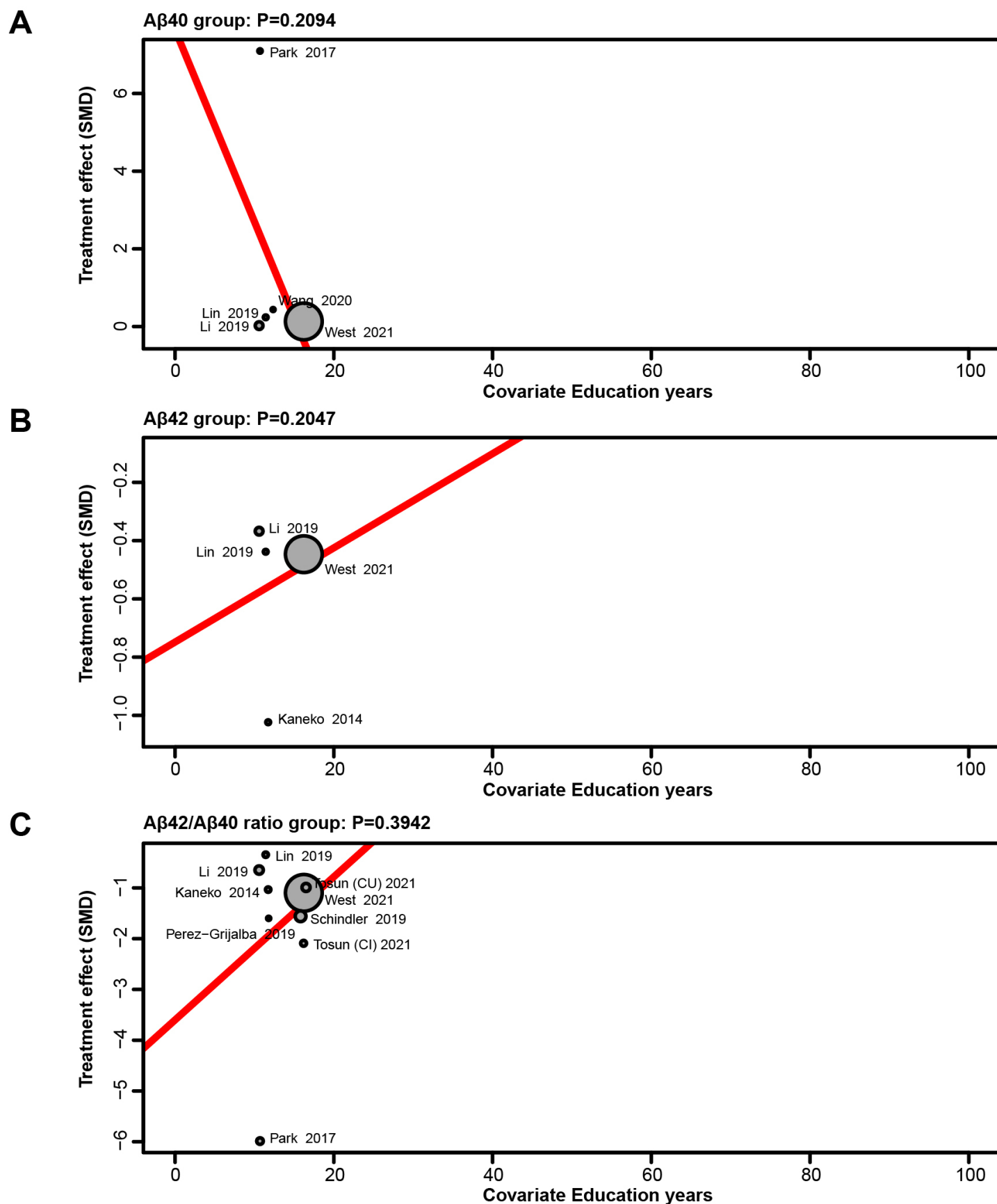

Supplement: Supplementary data [file jnnp-2021-327864supp004.pdf]

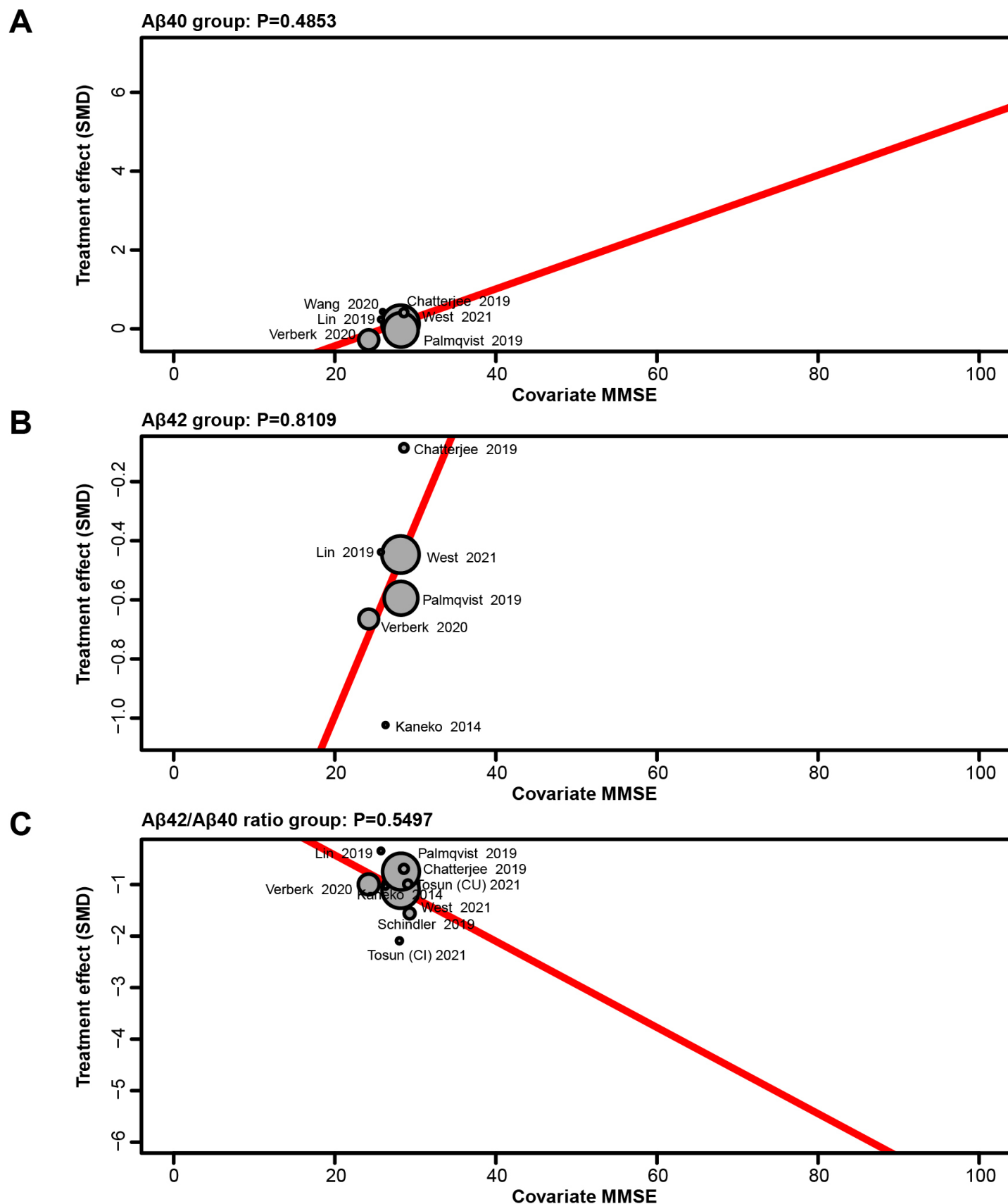

Supplement: Supplementary data [file jnnp-2021-327864supp005.pdf]

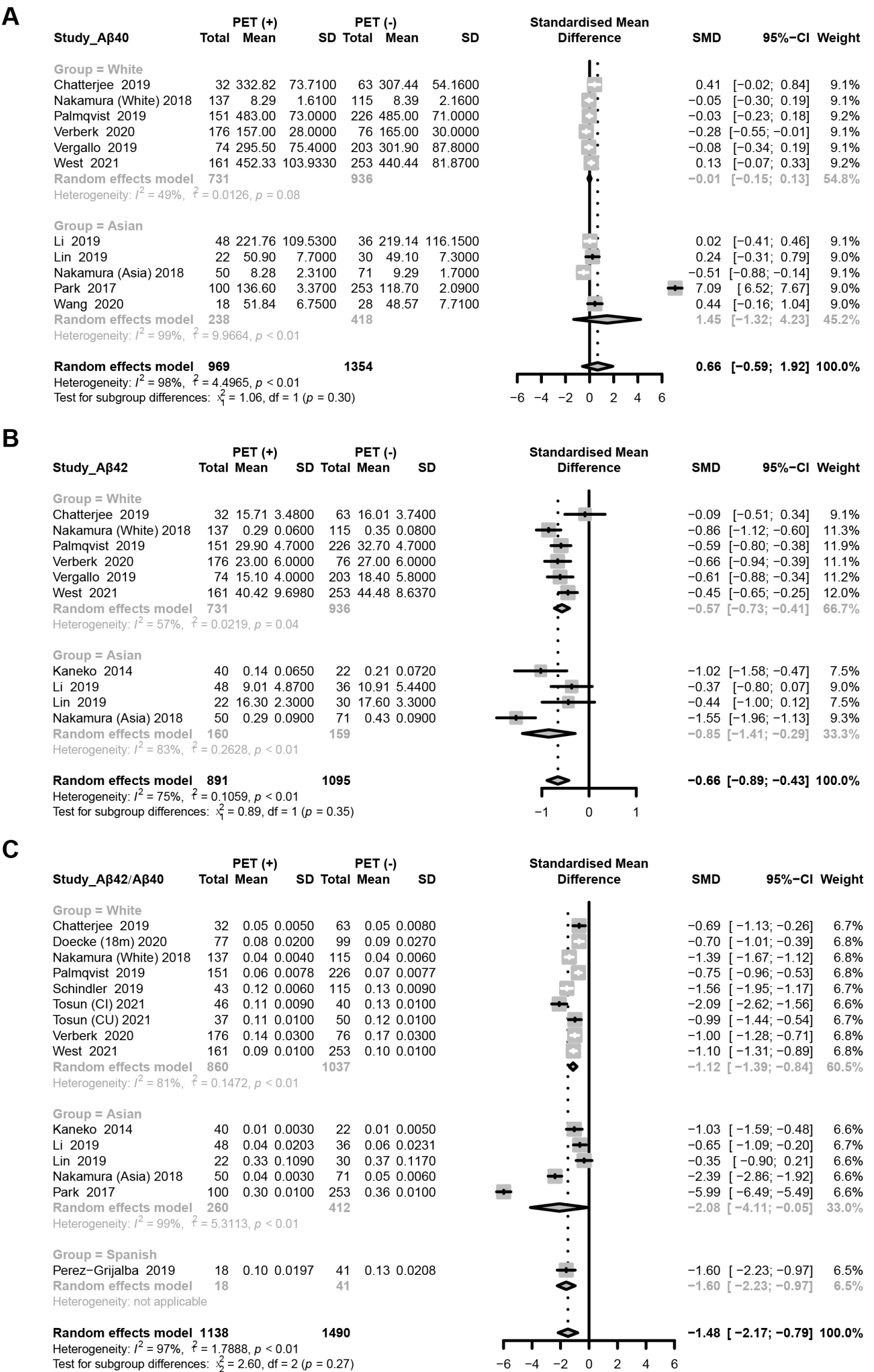

Supplement: Supplementary data [file jnnp-2021-327864supp006.pdf]

Supplement: Supplementary data [file jnnp-2021-327864supp007.pdf]

A

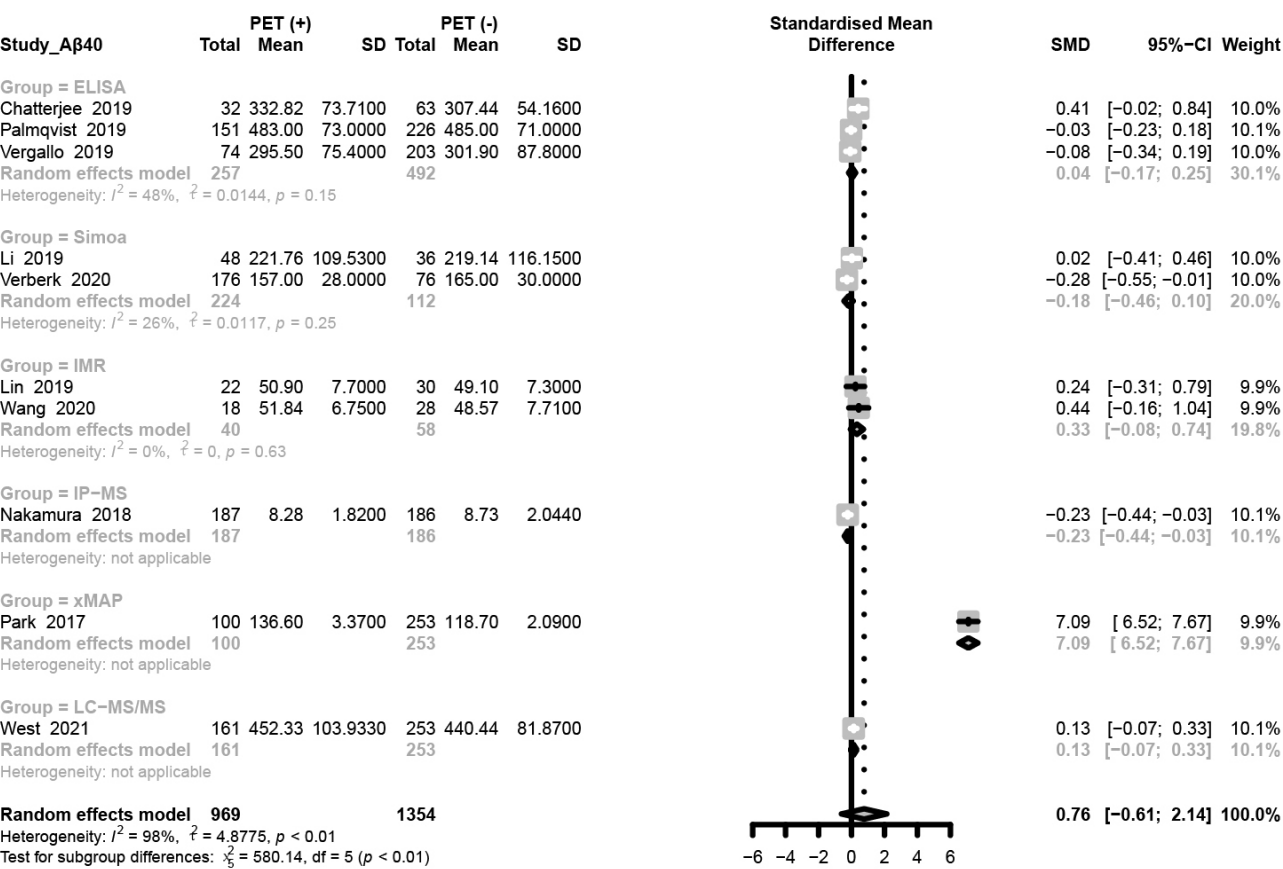

B

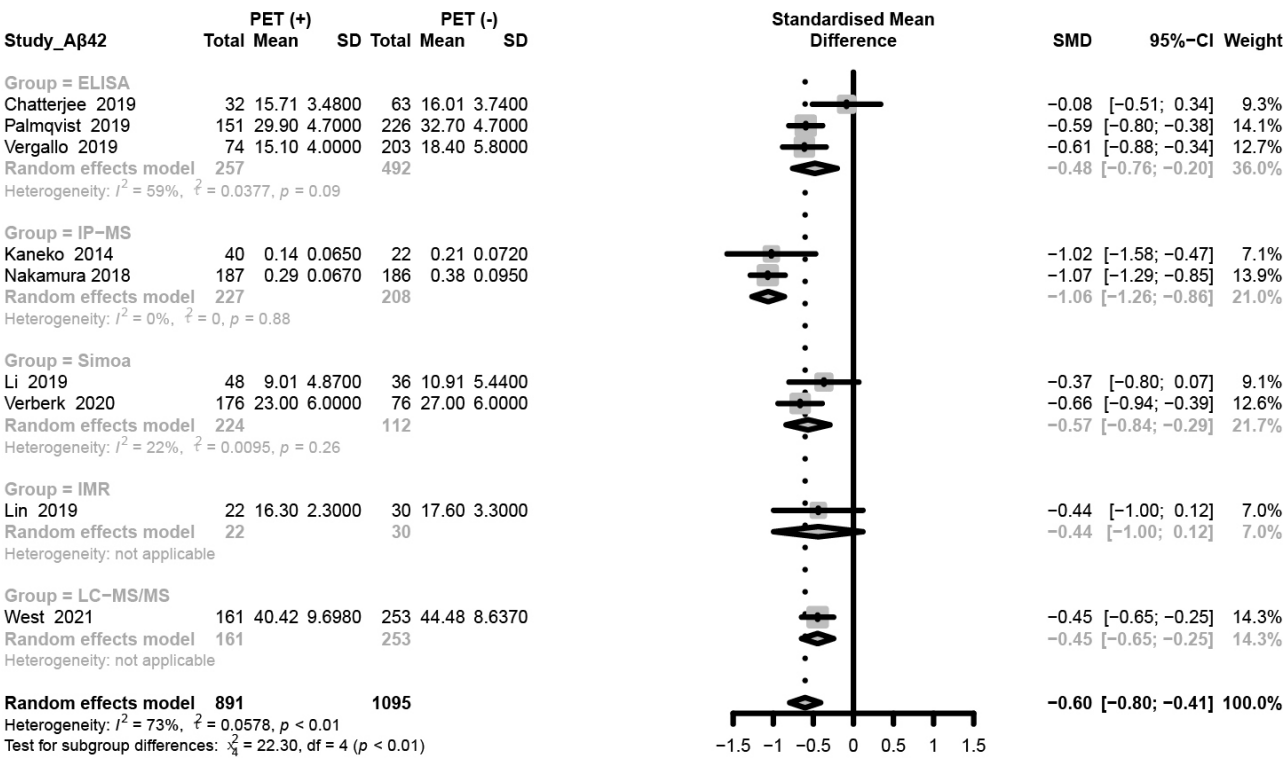

C

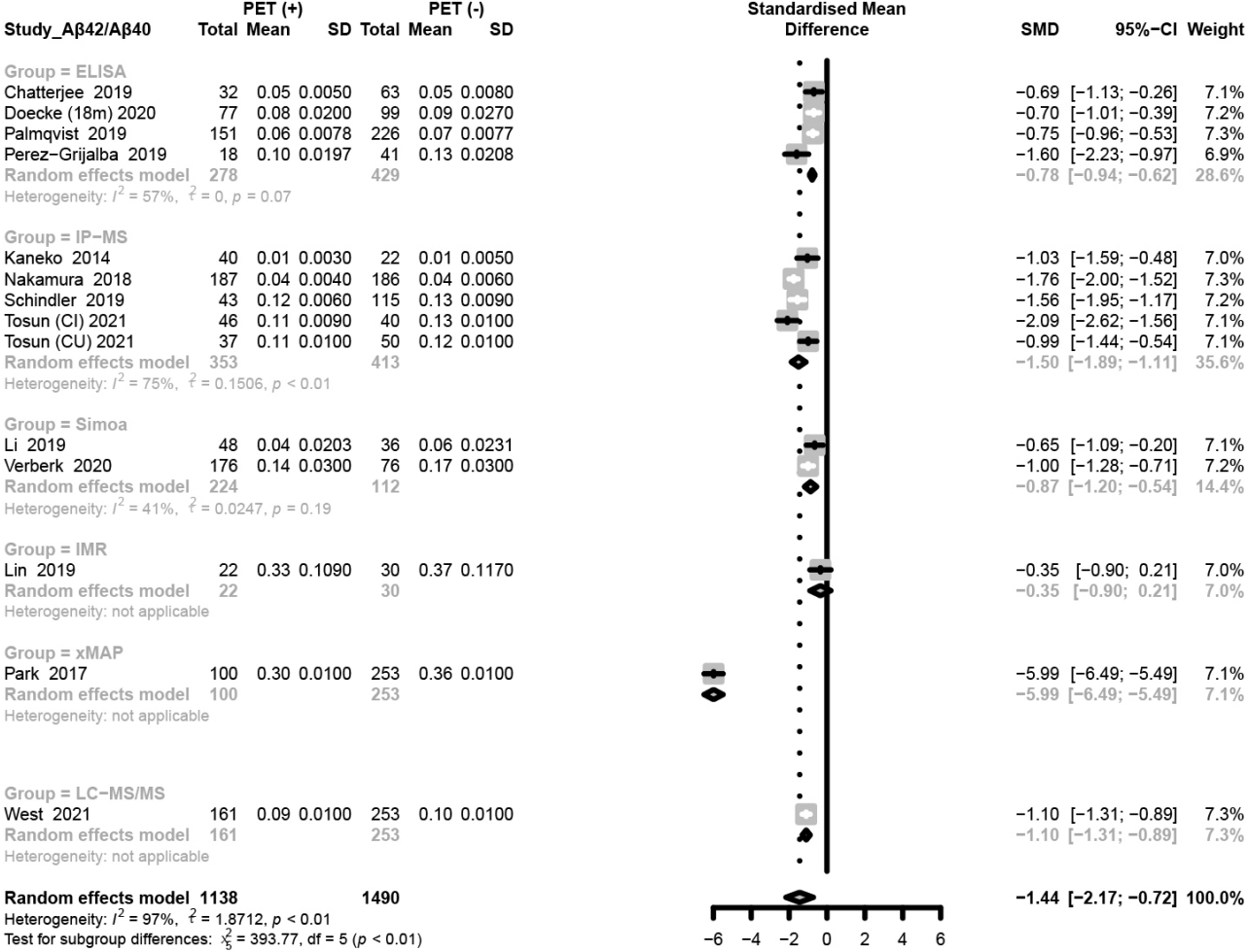

Supplement: Supplementary data [file jnnp-2021-327864supp008.pdf]
